# Primary bladder signet ring cell adenocarcinoma in a young male with Lynch Syndrome

**DOI:** 10.1016/j.eucr.2025.103223

**Published:** 2025-09-24

**Authors:** James Um, Aidan Kennedy, Christopher D. Jaeger, Nikta Rezakahn Khajeh, Paul Alan Horning, Reenal Patel, Kanika Arora, Adam J. Gadzinski

**Affiliations:** Department of Urology, Corewell Health William Beaumont University, Royal Oak, MI, United States of America

**Keywords:** Signet ring cell carcinoma, Bladder cancer, Lynch syndrome, Case report

## Abstract

A 32-year-old male with Lynch Syndrome and a history of colon cancer status post total proctocolectomy was found to have invasive high-grade carcinoma with signet ring cell morphology on tissue sampling of the bladder neck following transurethral resection of a bladder tumor (TURBT). The patient subsequently underwent an open cystoprostatectomy and completion proctectomy. Final pathology of the prostate, bladder, and bladder augment confirmed the diagnosis of signet ring cell adenocarcinoma (SRCC) of the bladder. Given its rarity, this case illustrates the challenges of managing an aggressive bladder cancer and underscores the importance of considering appropriate screening strategies in high-risk populations.

## 1 Introduction

Signet ring cell adenocarcinoma (SRCC) is a rare histologic subtype of adenocarcinoma that is characterized by abundant intracytoplasmic mucin that displaces the nucleus to the cell periphery, giving it the distinctive “signet ring” appearance. While SRCCs are more well known for being of gastrointestinal tract origin, there have also been reported cases in the breast, prostate, colon, and rarely the urinary bladder.^1^ Most cases of SRCC of the bladder are due to metastatic gastric, colonic, or breast malignancies, which makes cases of primary SRCC of the bladder all the more rare– accounting for only 1 %–4 % of all bladder cancers.^2^ It can occur across a broad age range but tends to increase in incidence after the age of 40, with a reported male-to-female ratio of approximately 2.7–3.2:1.^3^ This subtype of bladder cancer is highly malignant and often infiltrates the bladder wall without forming a discrete mass, leading to non-specific cystoscopic and radiologic findings, which can further delayed diagnosis. Additionally, SRCCs have a histological resemblance to benign and inflammatory changes, which can obscure their presence, especially in smaller biopsies.^4^^,^^5^ Early lesions typically show submucosal infiltrative growth, but symptoms tend to emerge only once the tumor has invaded the muscle layer—often preceded by chronic inflammation, cystitis, or glandular metaplasia.^6^^,^^7^ Because of this delayed presentation, prognosis is generally poor, with reported five-year survival rates as low as 27 %–30 %.^5^ Early radical cystectomy followed by adjuvant systemic chemotherapy may improve outcomes, and studies have identified T-stage as an independent risk factor for survival.^1^

## 2 Case report

Patient is a 32 year old male with a past medical history significant for Lynch syndrome, End-stage renal disease on hemodialysis and clean intermittent catheterization, myelomeningocele paraplegia, neurogenic bladder status post bladder augmentation and appendicovesicostomy as child, colon cancer status post total proctocolectomy with end colostomy (2017), who presented to the hospital on 12/18/2024 as a transfer from an outside hospital due to hemodynamic instability, abdominal pain and distention. Prior to presentation, the patient was under surveillance with tumor markers including CEA, CA-125, CA27-29, CA19-9, PSA, and AFP, and B-HCG, all of which were negative. A urethral catheter was placed and returned gross hematuria, which prompted the Urology department to conduct a cystoscopy on 12/30/2024 after the patient was stabilized.

An atypical frondular and papillary appearing tumor was visualized at the bladder neck with thick mucus and stones free floating in the lumen of the augmented bladder. The overall bladder anatomy was found to be completely distorted with no clear vision of any structure resembling a trigone or ureteral orifice. The aforementioned bladder neck tumor was resected and sent to pathology.

**Fig. 1:**
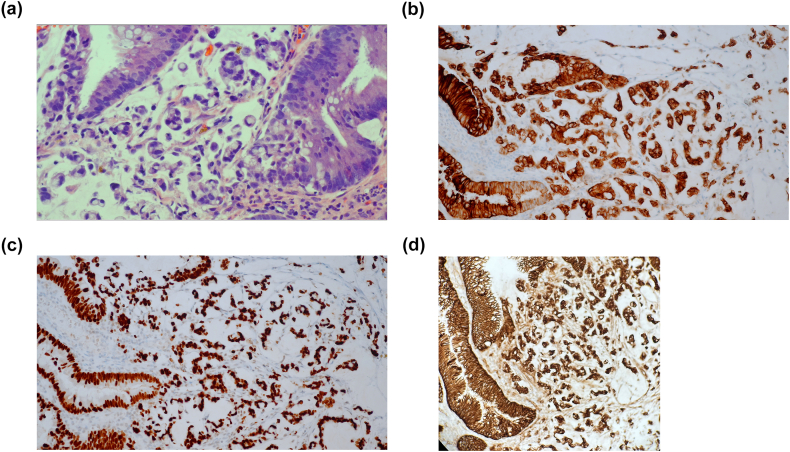
H&E image (40x) showing urothelial carcinoma with prominent signet ring cell morphology(a); immunohistochemistry reveals diffuse positivity for CK20(b), CDX2(c), membranous beta-catenin(d), supporting glandular differentiation.

**Fig. 2:**
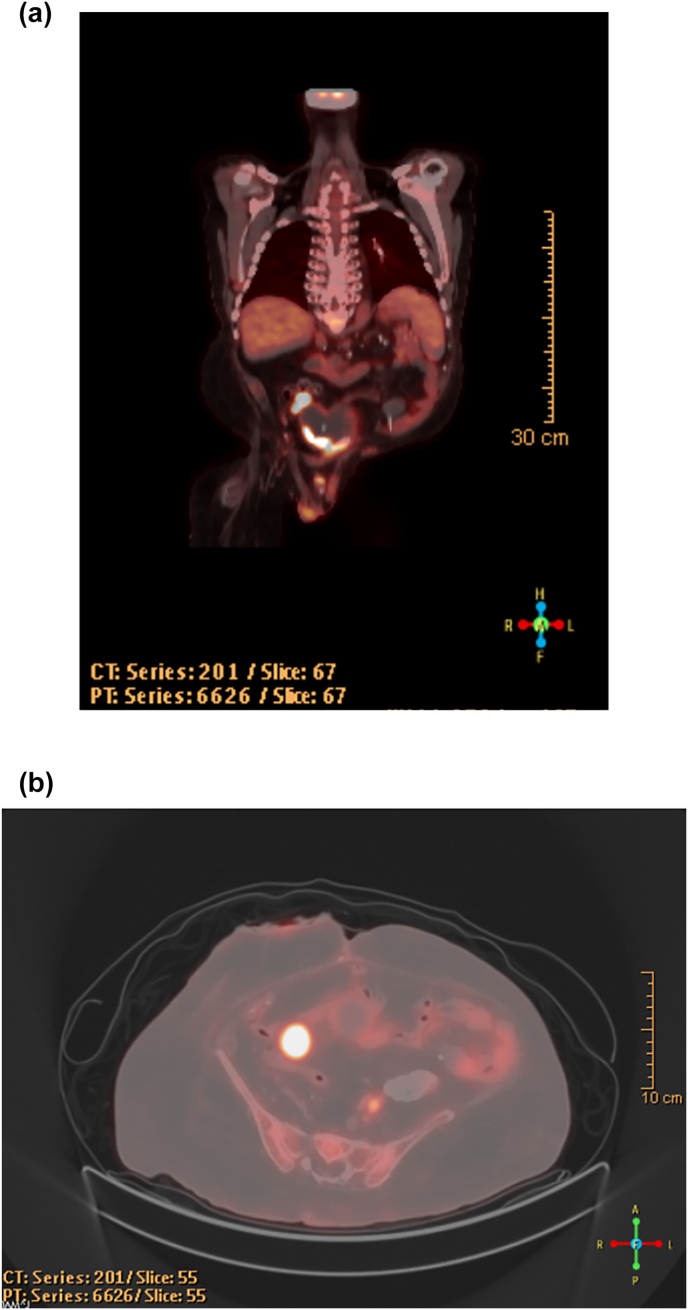
PET CT Images from 2/12/25 showing suspicious left-sided presacral lymph node. Coronal(a) and transverse views(b).

The pathology of the bladder tumor was revealed to be muscle invasive high-grade carcinoma with signet cell morphology (Fig. 1). A staging PET CT revealed likely involvement of a lymph node in the pre-sacral pelvis and possible lymph node involvement in the mesentery of the rectosigmoid colon. There was no evidence of distant metastasis (Fig. 2). The patient was discussed at the local institution's tumor board where it was felt reasonable to proceed with local surgical resection, as further delays may end up compromising risk of cancer control and radiation therapy was deemed to be a suboptimal oncological treatment. The patient underwent a radical cystoprostatectomy of the native bladder and augment with concurrent completion proctectomy. The resected prostate and bladder pathology was consistent with poorly differentiated mucinous adenocarcinoma with signet ring cells, consistent with bladder primary – deemed Stage IIIA (pT4a, Pn1, cM0). The resected colon showed no evidence of dysplasia or carcinoma.

Given lymph node involvement and aggressive nature of the malignancy, adjuvant radiation treatment was considered. The patient ultimately decided on palliative/comfort care. The patient passed away approximately 3 months following the radical surgery.

## 3 Discussion

Lynch syndrome increases the risk for a plethora of cancers, however guidelines for oncologic surveillance is dependent on organs at risk. The U.S. Multi-Society Task Force on Colorectal Cancer, American Gastroenterological Association and American Society for Gastrointestinal Endoscopy currently recommend colonoscopy every 1–2 years, beginning at the age of 20–25, or two to five years before the youngest case of colorectal cancer in the family, if the family member's cancer occurred before age 25. However, there is no current recommendation for routine bladder cancer screening in individuals with Lynch Syndrome. The American Gastroenterological Association, as part of the US Multi-Society Task Force on Colorectal Cancer states that urinary tract cancer, including bladder cancer, should be monitored with an annual urinalysis starting between age 30 to 35, though this is based on low quality evidence. Expert consensus states that Urinalysis is inexpensive and noninvasive, therefore, should be considered as routine for Lynch Syndrome patients.^8^^,^^9^

The relative risk of bladder cancer in Lynch Syndrome is 4–7.5 fold higher than the general population, with the prevalence being approximately 4.1 % in individuals with Lynch Syndrome.^9^ The primary bladder cancer type in patients with Lynch syndrome is urothelial carcinoma. The incidence of primary signet ring cell adenocarcinoma of the bladder is exceedingly rare, making up one to four percent of all bladder cancers.^2^ However, cases such as our case report highlight the need to adhere to current screening strategies and to consider advocating for comprehensive screening protocols. This patient's history of colon cancer diagnosis secondary to Lynch syndrome in his mid 20s could have prompted yearly urinalysis to screen for urothelial malignancies. Of note, the patient's *history of spina bifida and neurogenic bladder represents a potential confounding factor, as both are associated with an elevated risk of bladder cancer, often related to chronic catheterization*^10^*. However, the rare histology of signet ring adenocarcinoma is more consistent with a Lynch syndrome associated malignancy.*

Histologic mimicry between signet ring cell carcinoma of the colon and bladder is a well-recognized diagnostic challenge.^9^ In patients with Lynch Syndrome presenting with signet ring cell carcinoma of the bladder, the possibility of metastatic disease from a colorectal primary should be considered, especially given the rarity of primary bladder origin. Given the aggressive nature of these tumors, screening and early intervention is essential emphasizing the need for consensus-based, urologic screening recommendations.

## 4 Conclusion

We report a rare case of primary signet ring cell adenocarcinoma of the bladder in a young patient with Lynch Syndrome, history of myelomeningocele with bladder augmentation, and a prior history of colorectal cancer. Despite radical surgery, the patient's prognosis was poor due to the nature of this rare cancer subtype and regional metastasis. This case highlights the lethal nature of primary signet ring cell adenocarcinoma as well as the role that urologist can play in the screening and treatment of bladder cancer in patients with Lynch Syndrome.

## CRediT authorship contribution statement

**James Um:** Writing – original draft, Conceptualization. **Aidan Kennedy:** Writing – original draft. **Christopher D. Jaeger:** Writing – original draft. **Nikta Rezakahn Khajeh:** Writing – review & editing. **Paul Alan Horning:** Writing – review & editing. **Reenal Patel:** Writing – review & editing. **Kanika Arora:** Formal analysis. **Adam J. Gadzinski:** Writing – review & editing, Conceptualization.

## Funding

This research did not receive any specific grant from funding agencies in the public, commercial, or not-for-profit sectors.
